# The concerns of mechanical upper urinary tract obstruction in neurogenic lower urinary tract dysfunction: Beyond augmentation cystoplasty

**DOI:** 10.3389/fsurg.2023.1102272

**Published:** 2023-03-23

**Authors:** Zhonghan Zhou, Xuesheng Wang, Limin Liao

**Affiliations:** ^1^ Cheeloo College of Medicine, Shandong University, Department of Urology, China Rehabilitation Research Center, Beijing, China; ^2^ University of Health and Rehabilitation Sciences, Qingdao, China; ^3^ China Rehabilitation Science Institute, Beijing, China; ^4^ Beijing Key Laboratory of Neural Injury and Rehabilitation, Beijing, China; ^5^ School of Rehabilitation, Capital Medical University, Beijing, China

**Keywords:** augmentation uretero-enterocystoplasty, neurogenic bladder, upper urinary tract dilatation, upper urinary tract obstruction, vesicoureteral reflux

## Abstract

**Purpose:**

To evaluate the efficacy of augmentation uretero-enterocystoplasty (AUEC), a modified surgical procedure that focuses the mobilization of the ureter and the necessity of ureteroplasty in a series of neurogenic lower urinary tract dysfunction (NLUTD) patients with mechanical upper urinary tract obstruction (mUUTO).

**Methods:**

We retrospectively reviewed the medical records of NLUTD patients who underwent an AUEC from 2005 to 2022. mUUTO was diagnosed by preoperative bladder drainage, magnetic resonance urography (MRU), and isotope renography. Upper urinary tract dilatation (UUTD) was evaluated using MRU with the Liao MRU-UUTD system.

**Results:**

A total of 58 patients and 103 ureters were analyzed. Improvement in maximum bladder capacity (from 79.0 [41.3–163.8] to 500.0 [450.0–597.5] ml, *P* < 0.001), maximum detrusor pressure (from 32.0 [13.0–50.8] to 5.5 [4.0–10.0] cmH_2_O, *P* < 0.001) and bladder compliance (from 6.5 [3.0–11.9] to 50.1 [37.5–65.0] ml/cmH_2_O, *P* < 0.001), and stabilization of serum creatine (93.4 [73.0–142.7] to 94.9 [72.2–148.7] μmol/L, *P* = 0.886) were observed. The proportion of high-grade UUTD was significantly reduced after the surgery (92.3% vs. 13.5%, 92.1% to 9.8%, *P* < 0.001), and the typical imaging signs of preoperative obstruction disappeared.

**Conclusion:**

Beyond traditional augmentation cystoplasty, more attention should be paid to the relief of mUUTO and mobilization of the ureter in NLUTD patients.

## 1. Introduction

Neurogenic lower urinary tract dysfunction (NLUTD) is a major problem in patients with central or peripheral nerve disorders and may result in debilitating symptoms and serious complications (1, 2). Increased bladder pressure, usually caused by detrusor overactivity, detrusor sphincter dyssynergia, or impaired bladder compliance, is among the most devastating problems for NLUTD patients. Increased pressure applied to the upper urinary tract and cause ureteral dilation, megaureter, and hydronephrosis, which are termed upper urinary tract dilatation (UUTD) (3, 4). UUTD can be subclassified into one of four categories based on the cause: obstructed UUTD (oUUTD), refluxing UUTD (rUUTD), refluxing-obstructed UUTD (roUUTD), and nonobstructed-nonrefluxing UUTD (nUUTD). UUTD is quite common in NLUTD patients, and preserving upper urinary tract function is extremely important in the management of NLUTD.

Augmentation cystoplasty (AC) and/or ureteral anti-reflux reimplantation (UARI) is a widely accepted reconstructive procedure used when conservative treatment fails (5–8). Previously, urologists mainly focused on the amelioration of bladder function and the elimination of vesicoureteral reflux (VUR) (9, 10). However, many NULTD patients presented with mechanical upper urinary tract obstruction (mUUTO). This is especially common in China and other developing countries, where patients have delayed treatment initiation, inappropriate bladder management, and untimely and unstandardized treatment (4). The oUUTD or roUUTD cannot resolve spontaneously after using bladder drainage or relieving intravesical pressure. It is not sufficient to perform AC and/or UARI for these patients. Ureteroplasty, including the complete relief of mUUTO and tailoring/shortening of the ureter, together with AC/UARI, is essential for the preservation of upper urinary tract function. Therefore, we introduced a modified surgical procedure for NLUTD patients, augmentation uretero-enterocystoplasty (AUEC), which emphasizes the repair and reconstruction of the upper urinary tract (11). We analyzed a series of NLUTD patients with mUUTO, elaborated the surgical procedure, and analyzed its efficacy in this population.

## 2. Patients and methods

### 2.1. Patients

We reviewed the medical records of NLUTD patients who underwent AUEC at the China Rehabilitation Research Center from 2008 to 2021. The study was approved by the Ethics Committee of the China Rehabilitation Research Center (No. 2017-003-1). General clinical information, laboratory data, and imaging records of the patients were collected. The maximum bladder capacity (MBC), maximum detrusor pressure (MDP), and bladder compliance were obtained from video urodynamics (VUD). VUR was graded based on the international reflux grading system (12). UUTD was evaluated by magnetic resonance urography (MRU) with the Liao MRU-UUTD system. The novel system graded UUTD (Grades 0 to 4) according to the dilation of the central renal complex, tortuous and dilation of the ureter, and the thickness of renal parenchyma from the coronal, transverse, and three-dimensional MRU reconstruction images, which we described in previous publications (3, 4). mUUTO was assessed using MRU and isotope renography (13). Typical imaging findings includes external compression, stricture, and angulation of the ureter, with the proximal ureter dilation above the obstruction; the failure of UUTD relief after sufficient peroperative bladder drainage for 3 month suggests the diagnosis of mUUTO. All patients underwent isotope renography and diuresis experiments to detect mechanical or adynamic obstructions based on perfusion time-activity curves.

### 2.2. Surgical technique

**Figure 1:**
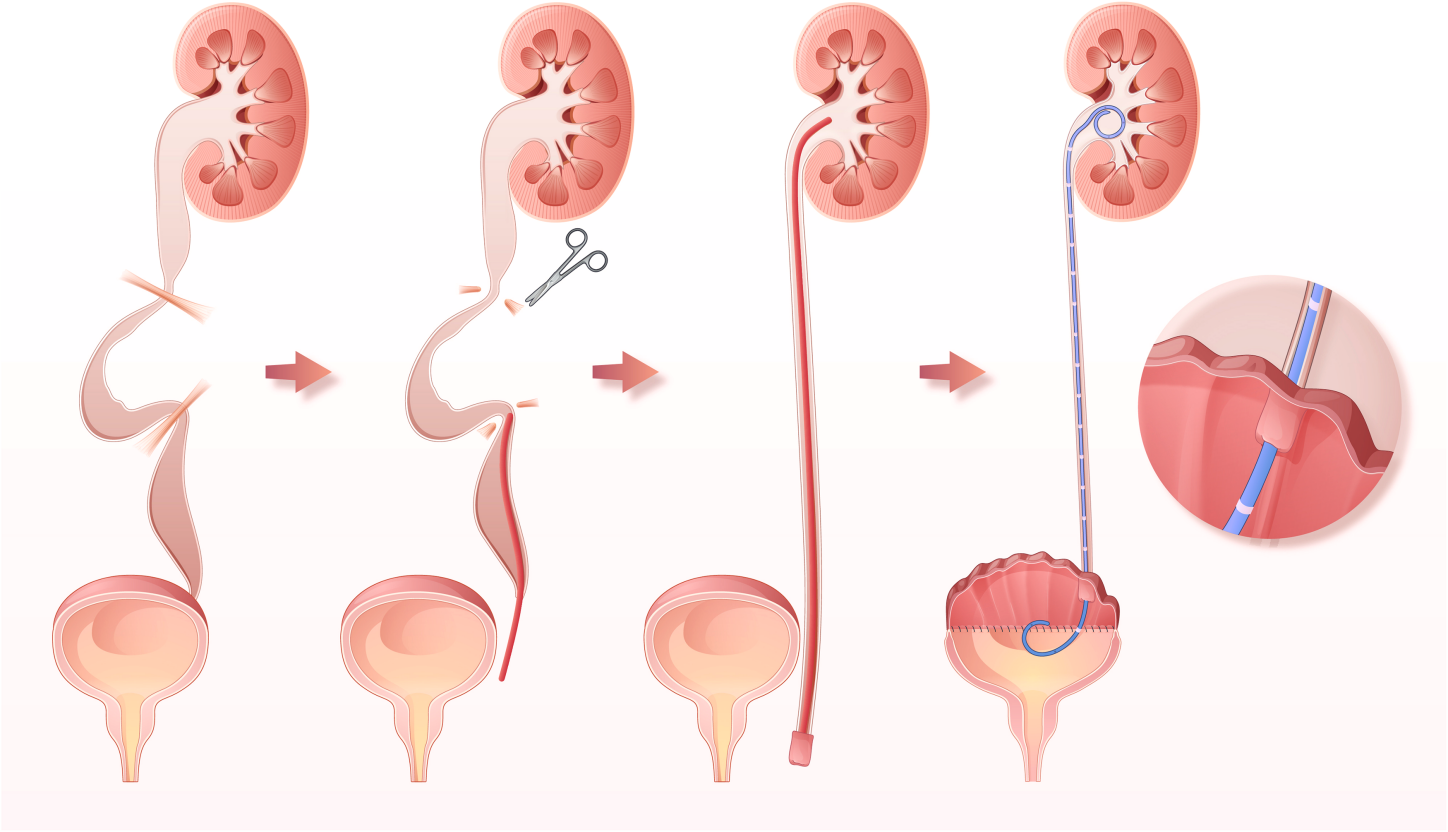
Illustrations of AUEC. (**A**) UUTD characterized as external compression, stricture, and angulation of the ureter, with proximal ureter tortuosity and dilation above the obstruction. (**B**) Releasing of the tortuosity knots or constricting fibrous bands, straightening, shorting, and tailoring of the ureter. (**C**) Straightening, shorting, and tailoring of the ureter. (**C**) The successful mobilization of the ureter, indicated by the presence of abundant urine drainage from pelvis. (**D**) Intravesical mobilization of the terminal ureter, with subsequent reimplantation on the neobladder through a new hiatus and Kock nipple valve.

AUEC was performed *via* a low-midline incision with the patient in the supine position. About 25 to 30 centimeters of ileum or sigmoid colon segment was harvested and detubularized along the mesenteric border. The intestinal segment was sutured in a “U” shape to form a substitutable patch. The urinary bladder was incised longitudinally. Ureterolysis was performed from the level of the vesicoureteral junction (VUJ) to the ureteropelvic junction (UPJ) (Figure 1A). The ureter was detached from the VUJ, and any tortuosity knots or constricting fibrous bands were released. Mobilization and straightening of the ureter were confirmed by retrograde intubation of a 10G to 12G urinary catheter. Any obstruction along the ureter prevented the catheter from migrating superiorly, indicating the presence of a knot or fibrous bands (Figure 1B). The presence of abundant urine drainage showed that the catheter reached the UPJ level, suggesting successful mobilization of the ureter (Figure 1C). In cases with severe ureteral tortuosity or megaureter, tailoring/shortening was performed to reduce the ureter's length and diameter. One or two 7G double-J catheters were then inserted into the ureter (Figure 1D). The UARI procedure was performed by intravesical mobilization of the terminal ureter, with subsequent reimplantation on the native bladder or neobladder through a new hiatus and Kock nipple valve (Figure 1E). Then the bladder was anastomosed to the bowel patch, and a suprapubic catheter was placed before completion of the anastomosis. There are several points to be noted during the operation. First, protect the blood supply of the ureters as much as possible. Second, remove the hyperplastic scars and fibrotic ureters as much as possible, as long as the length of ureter is enough. Third, a hemi Kock nipple valve of 1 cm in length is made, and should be matched with the size of the reimplantation channel. Forth, the double-J catheters should be retained for at least 4 weeks, or 12 weeks in some cases.

### 2.3. Statistical analysis

Statistical analysis was performed using R (version 4.1.1; The R Foundation, Vienna, Austria). Variables with a normal distribution were expressed as mean ± standard deviation [SD] and evaluated using a paired Student's *t*-test. Asymmetrically distributed data were expressed as the median [Q1–Q3] and evaluated with a Wilcoxon signed-rank test. Categorical variables are presented as numbers or percentages and analyzed with a chi-square test or Fisher's exact probability method. All statistical tests were two-sided with an alpha of 0.05.

## 3. Results

### 3.1. Patient characteristics

Fifty-eight patients were analyzed in this study. Spinal cord injury, myelomeningocele, tethered cord syndrome, and spinal bifida were the most common causes of NLUTD. The median disease course was 12.0 [4.0–18.0] years, and median follow-up was 29.9 [19.7–42.0] months. A total of 103 ureter units with mUUTO were analyzed. All ureters had mUUTO diagnosed on isotope renography and diuresis experiments, in combination with typical MRU imaging findings and failure of UUTD relief through sufficient preoperative bladder drainage for 3 month. The median time to peak in perfusion time-activity curves was 15.7 [7.11–21.05] min. A total of 80.88% of ureters did not reach 50% activity, and the 20-minute residual rate was 80.2% [64.5%–93.1%] after administration of diuretics. Of 103 ureter units, 72 (69.90%), 23 (22.33%), 8 (7.77%) were classified as Grade 4, Grade 3, and Grade 2, respectively. Among them, 51 (49.51%) were accompanied by VUR (roUUTD), and 52 (50.49%) were classified as oUUTD. Patients' clinical characteristics are summarized in Table 1.

**Table 1:** The clinical characteristics of patients.

Parameters	Value
No. of patients	58
Age	26.4 ± 14.6
Gender	
Male	42 (72.41%)
Female	16 (27.59%)
Disease course (years)	12.0 [4.0–18.0]
Follow-up duration (months)	29.9 [19.7–42.0]
Etiology	
Spinal cord injury	12 (20.69%)
Myelomeningocele	12 (20.69%)
Tethered cord syndrome	8 (13.79%)
Spinal bifida	8 (13.79%)
Lumbosacral tumor	4 (6.90%)
Unclear	14 (24.14%)
Preoperative treatments	
Anticholinergics	58 (100.00%)
Botulinum toxin A	3 (5.17%)
Sacral neuromodulation	1 (1.72%)
Preoperative urine drainage pattern	
Spontaneous or assisted emptying	31 (53.45%)
Indwelling catheterization	12 (20.69%)
Suprapubic cystostomy	7 (12.07%)
Nephrostomy	3 (5.17%)
Clean intermittent catheterization	5 (8.62%)
Postoperative urine drainage pattern	
Clean intermittent catheterization	58 (100.00%)
Isotope renogram parameters	
Time to peak (minutes)	15.7 [7.11–21.05]
20-minute residual rate	80.2% [64.5%–93.1%]
GFR of differential kidney (ml/min/1.73 m^2^)	35.7 [22.7–49.3]
UUTD^a^ (4/3/2)	72/23/8
VUR^a^ (V/IV/III/II/I/0)	19/14/14/3/1/52
Maximum bladder capacity (ml)	79.0 [41.3–163.8]
Maximum detrusor pressure (cmH_2_O)	32.0 [13.0–50.8]
Bladder compliance (ml/cmH_2_O)	6.5 [3.0–11.9]
Serum creatine (μmol/L)	93.4 [73.0–142.7]
Gut segment (sigmoid/ileum)	55/3

GFR, glomerular filtration rate; VUR, vesicoureteral reflux; UUTD, upper urinary tract dilatation.

^a^ Ureteric units.

### 3.2. Improvement in VUD parameters

**Figure 2:**
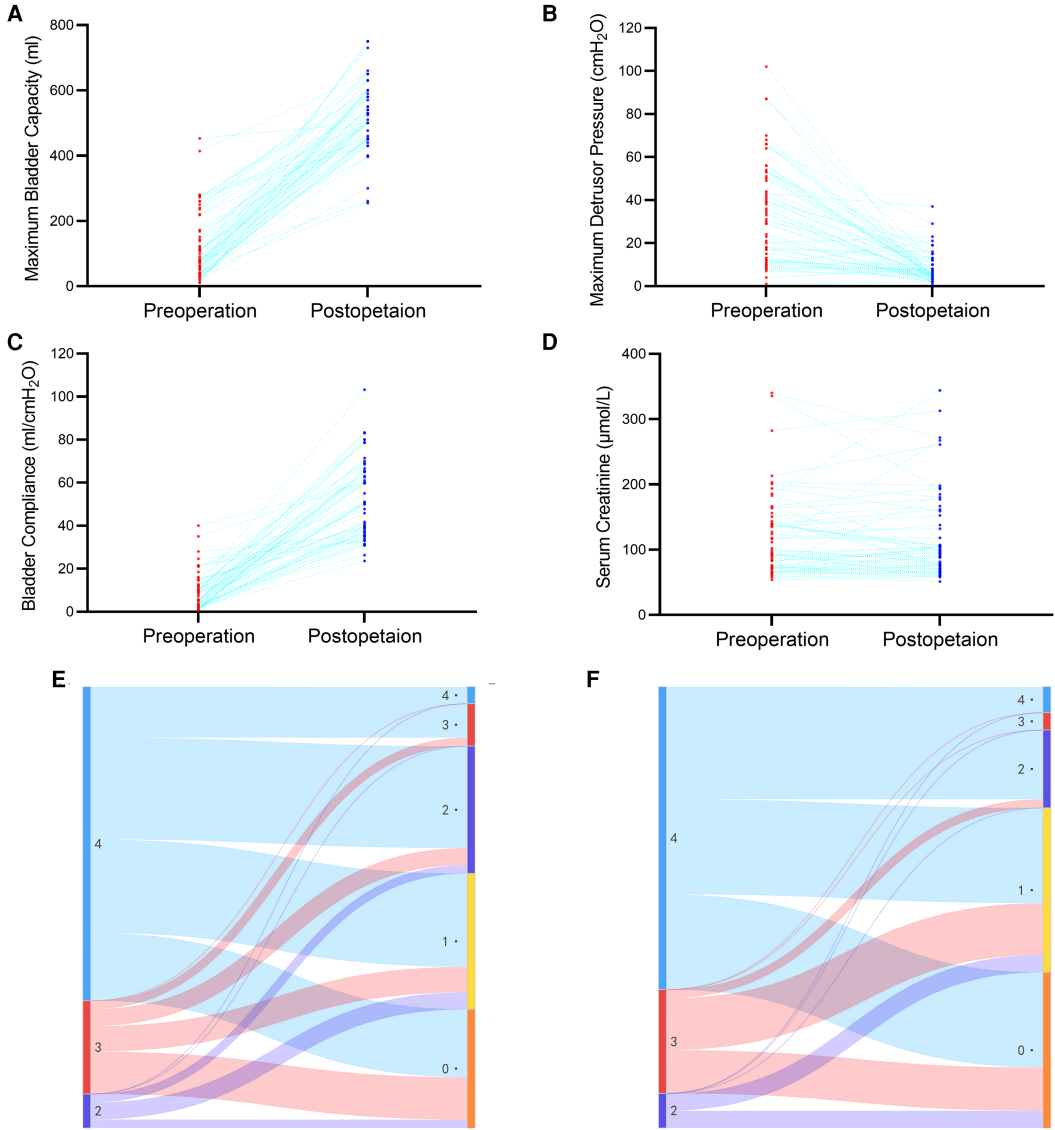
Changes in maximum bladder capacity (**A**), maximum detrusor pressure (**B**), bladder compliance (**C**), serum creatine (**D**), UUTD grades in the oUUTD (**E**) and roUUTD (**F**) group before and after surgery.

A significant improvement in MBC was observed after surgery (from 79.0 [41.3–163.8] ml to 500.0 [450.0–597.5] ml, *P* < 0.001, Figure 2A). MDP during the storage phase also decreased significantly (from 32.0 [13.0–50.8] cmH_2_O to 5.5 [4.0–10.0] cmH_2_O, *P* < 0.001, Figure 2B). Moreover, a significant difference in bladder compliance was also observed (from 6.5 [3.0–11.9] ml/cmH_2_O to 50.1 [37.5–65.0] ml/cmH_2_O, *P* < 0.001, Figure 2C). All patients received acceptable MBC and performed clean intermittent catheterization after surgery.

### 3.3. Stabilization of renal function

The preoperative serum creatinine was 93.4 [73.0–142.7] μmol/L, and the GFR of the differential kidney was 35.7 [22.7–49.3] ml/min/1.73 m^2^. No statistical difference in serum creatinine [94.9 (72.2–148.7) µmol/L] was observed during postsurgical follow-up (*P* = 0.886, Figure 2D), indicating stabilization of renal function.

### 3.4. Improvement in VUR and UUTD

**Figure 3:**
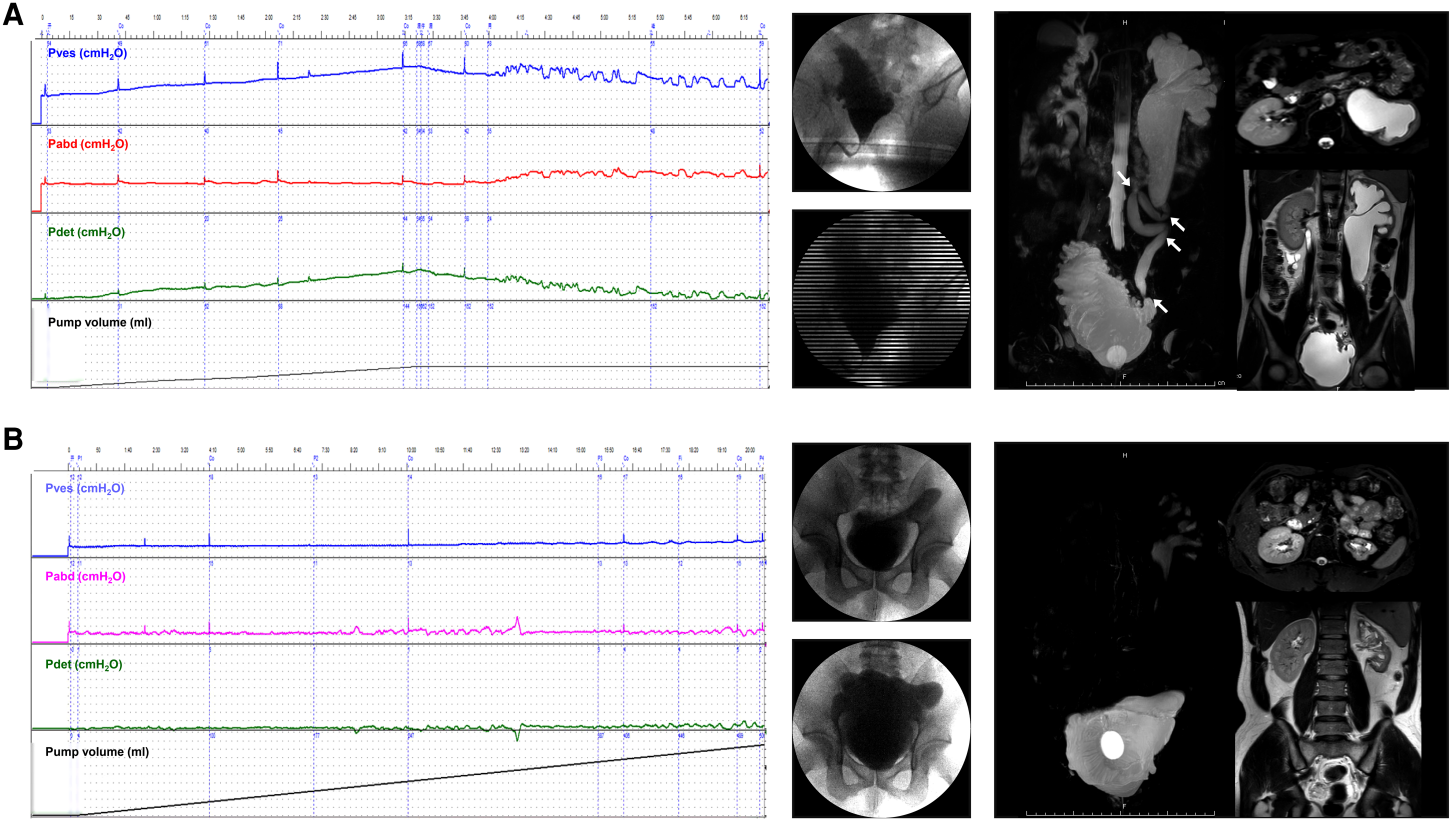
Improvement of VUD parameters and UUTD grades in a patient with preoperative oUUTD. (**A**) A 35-year-old man was admitted to our hospital with dysuria since birth. The serum creatinine was 96.2 µmol/L, and isotope renography showed a glomerular filtration rate of 13.0 ml/min (left) and 67.0 ml/min (right). VUD showed decreased bladder capacity (151.0 ml) and bladder compliance (5.8 ml/cmH_2_O), with a maximum detrusor pressure of 35.0 cmH_2_O. No VUR was observed; however, incontinence was observed at a volume of 151.0 ml (Pdet: 35.0 cmH_2_O). MRU showed a fully dilated renal pelvis, fluid-filled calices throughout the left kidney, and a tortuous, angulated ureter (Grade 4) with external compression and proximal ureter dilation above the obstruction that could not be relieved using a preoperative indwelling catheter for sufficient bladder drainage. The arrows indicate ureteral stenosis and tortuosity caused by fibrous band formation or peri-ureteral tissue adhesions, which were confirmed during surgery. All tortuosity knots or constricting fibrous bands were released, and mobilization of the ureter was confirmed using retrograde intubation of a 10 G urinary catheter. The excess terminal ureter was removed, and UARI was performed on the neobladder through a new hiatus and a Kock nipple valve. (**B**) The patient was admitted to our hospital 4.5 years after AUEC for re-examination. The serum creatinine was 103.5 μmol/L. The VUD showed a bladder capacity of 510.0 ml and a maximum detrusor pressure of 5.0 cmH_2_O. No VUR was detected. MRU showed relieved UUTD (Grade 0), indicating successful relief of mUUTO and mobilization of the ureter. VUD, video urodynamics; UUTD, upper urinary tract dilation; oUUTD, obstructed upper urinary tract dilation; VUR, vesicoureteral reflux; MRU, magnetic resonance urography; UARI, ureteral anti-reflux reimplantation; AUEC, augmentation uretero-enterocystoplasty; mUUTO, mechanical upper urinary tract obstruction.

A total of 52 ureter units were analyzed in the oUUTD group based on isotope renography and MRU imaging, including 37 (71.2%) of Grade 4, 11 (21.1%) of Grade 3, and 4 (7.7%) of Grade 2. A significant improvement in UUTD was observed after surgery, with 2 (3.8%) of Grade 4, 5 (9.7%) of Grade 3, 15 (28.8%) of Grade 2, 16 (30.8%) of Grade 1, and 14 (26.9%) of Grade 0. The rate of high-grade UUTD was significantly decreased after surgery (92.3% to 13.5%, *P* < 0.001, Figure 2E, Figure 3).

**Figure 4:**
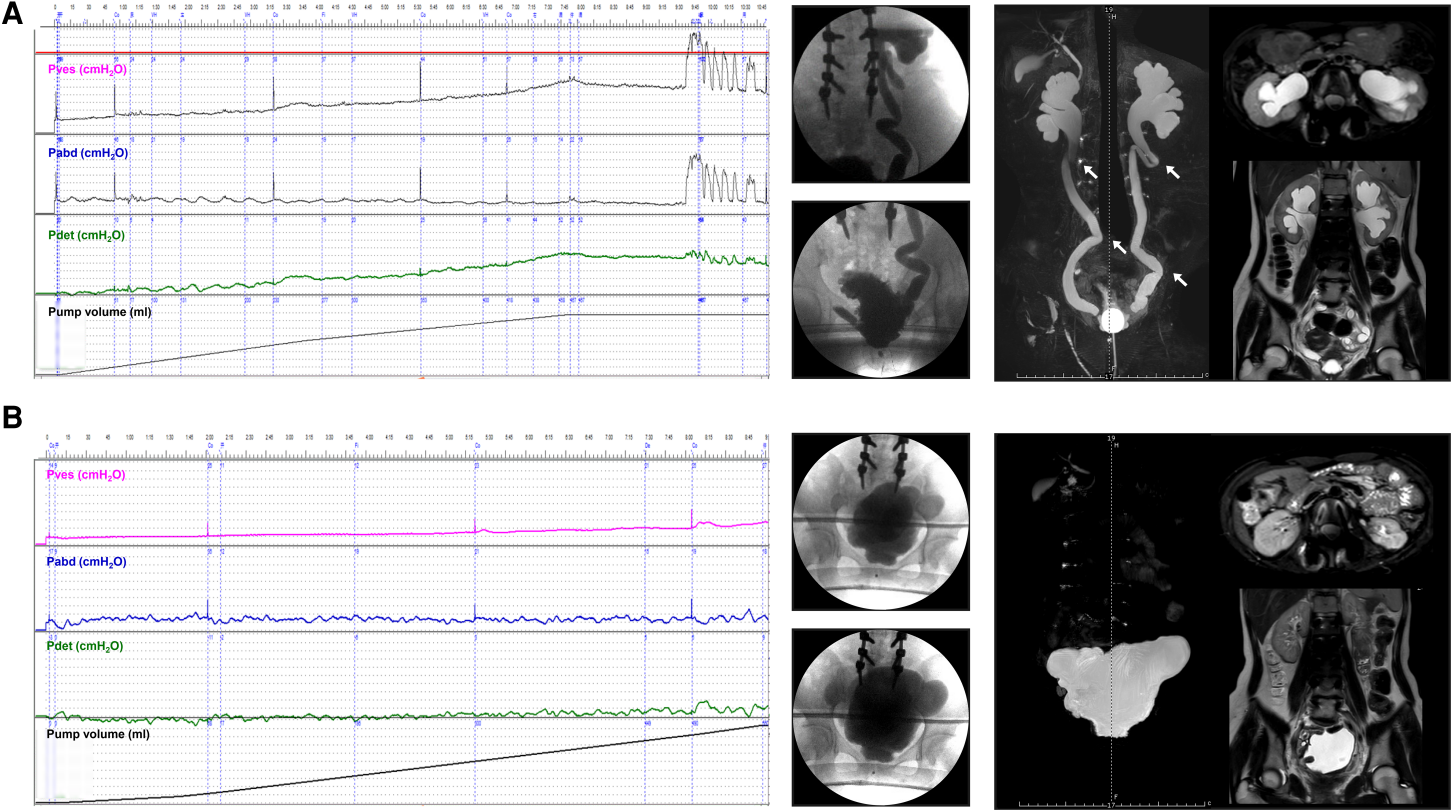
Improvement of VUD parameters and UUTD grades in a patient with preoperative roUUTD. (**A**) A 17-year-old girl was admitted to our hospital with urinary frequency and urgency since birth. The serum creatinine was 156.0 μmol/L, and isotope renography showed a glomerular filtration rate of 36.3 ml/min (left) and 42.5 ml/min (right). No decline in the perfusion time-activity curve was observed after administration of diuretics, suggesting the presence of mUUTO. Neither ureter reached 50% activity, and 20-minute residual rates were 84.6% and 94.2%, respectively. VUD showed decreased bladder capacity (79.0 ml) and bladder compliance (10.2 ml/cmH_2_O), with a maximum detrusor pressure of 52.0 cmH_2_O. Left and right VUR were observed at volumes of 79.0 ml (Pdet: 5.0 cmH_2_O, VUR grade: IV) and 262.0 ml (Pdet: 44.0 cmH_2_O, VUR grade: I), respectively. MRU showed a fully dilated renal pelvis, fluid-filled calices throughout the kidney, and tortuous and angulated ureters (Grade 3) that could not be relieved by a preoperative indwelling catheter for sufficient bladder drainage. The arrows indicate ureteral stenosis and tortuosity caused by fibrous band formation or peri-ureteral tissue adhesions. All the tortuosity knots or constricting fibrous bands were released, and mobilization of the ureter was confirmed by retrograde intubation of a 10 G urinary catheter. The excess terminal ureter was removed, and UARI was performed on the neobladder through a new hiatus and a Kock nipple valve. (**B**) The patient was admitted to our hospital 3 years after AUEC for re-examination. The serum creatinine decreased to 74.1 μmol/L. The VUD showed a bladder capacity of 560.0 ml and a maximum detrusor pressure of 5.0 cmH_2_O. No VUR was detected. MRU showed improved UUTD (Grade 0), suggesting the successful relief of mUUTO and reconstruction of the anti-reflux mechanism. VUD, video urodynamics; UUTD, upper urinary tract dilation; roUUTD, refluxing-obstructed upper urinary tract dilation; VUR, vesicoureteral reflux; MRU, magnetic resonance urography; UARI, ureteral anti-reflux reimplantation; AUEC, augmentation uretero-enterocystoplasty; mUUTO, mechanical upper urinary tract obstruction.

A total of 51 ureter units had both VUR and mUUTO. In the roUUTD group, there were 19 ureter units (37.3%) of VUR V, 14 (27.4%) of Grade IV, 14 (27.4%) of Grade III, 3 (5.9%) of VUR II, and 1 (2.0%) of Grade I. The preoperative UUTD level was Grade 4 in 35 (68.6%), Grade 3 in 12 (23.5%), and Grade 2 (7.9%) in 4. After reconstructing the anti-reflux mechanism and releasing the ureter obstruction, all the VUR resolved, and the rate of high-grade UUTD declined from 92.1% to 9.8% (*P* < 0.001, Figure 2F, Figure 4), including 3 (5.9%) ureter units of Grade 4, 2 (3.9%) of Grade 3, 9 (17.6%) of Grade 2, 19 (37.3%) of Grade 2, and 18 (35.3%) of Grade 0.

## 4. Discussion

Previously, urologists focused only on reconstruction of the bladder and elimination of VUR for NLUTD patients (5, 6). However, many patients have severe UUTD without VUR. This is especially common in patients with delayed treatment initiation and unstandardized bladder management (4). The appearance of oUUTD complicates treatment protocols because it is not sufficient to decrease intravesical pressure to eliminate hydronephrosis and preserve renal functions. Beyond AC, more attention should be paid to the relief of obstruction and mobilization of the ureter in NLUTD patients.

We analyzed a series of patients with intractable NLUTD characterized as oUUTD or roUUTD at initial evaluation. For the treatment of primary congenital megaureter in pediatric urology, there are debates about whether dilation is associated with increased risks of high serum creatine, which allows watchful waiting or conservative treatment (14, 15). However, the presence of UUTO and related oUUTD indicate advanced stage in NLUTD patients. It is often associated with impaired renal function and cannot spontaneously resolve. These patients need more aggressive surgical intervention. In these patients with obstruction above the bladder, it is not sufficient to perform AC and/or UARI without releasing the mechanical obstruction (11). For them, severe UUTD cannot be relieved by preoperative bladder drainage (indwelling catheter or suprapubic cystostomy), which indicates the existence of UUTO. In some NLUTD patients without proper bladder management, an indwelling catheter may be associated with recurrent urinary tract infection and chronic inflammation, which may conversely lead to UUTO and progression of UUTD. Here, the value of uretero-enterocystoplasty beyond AC is revealed; mobilization of the ureter should be more concerned and the procedure of ureteroplasty is indispensable. It is essential to completely release tortuous knots and constricting fibrous bands and perform ureter tailoring/shorting to restore the ureter's normal diameter and length. Concomitant UARI is usually performed to eliminate the obstruction and reconstruct the anti-reflux mechanism, especially for patients with high-grade, low-pressure VUR (9). However, it is inappropriate to merely perform ureteroplasty without AC. Unlike primary megaureter or UPJ obstruction, the treatment of secondary UUTD should be directed toward the cause. Failure to decrease intravesical pressure and ameliorate bladder compliance will lead to disease progression, UUTD persistence, and the deterioration of renal function.

The improved VUD parameters, including the MBC, MDP, and bladder compliance, are consistent with previously published data for AC (16–18). The results also demonstrated stable levels of serum creatine during follow-up, indicating preserved renal function. The proportion of high-grade UUTD was significantly reduced after surgery, and the typical preoperative obstruction on imaging resolved. Although postoperative isotope renography data were missing, the amelioration of UUTD in MRU imaging suggests successful mobilization of the ureter and relief of mUUTO. Residual low-grade UUTD is observed in some patients due to impaired peristalsis; however, 12 ureter units (11.6%) had postoperative high-grade UUTD. UUTD caused by tissue edema of the anastomosis can regress or subside spontaneously after adequate drainage of urine from the indwelling catheter and anti-infection treatment; however, persistent UUTD occurs in some cases. Residual or new-onset UUTD may result from vesicoureteral anastomosis stenosis or fibrous band formation in retroperitoneal tissue after surgery, which may be caused by interruption of the ureter blood supply or chronic inflammation exudation (11, 19–21). In such cases, conservative management or reoperation should be used based on changes in UUTD and renal function over follow-up.

We previously introduced a UUTD grading system using MRU in patients with NLUTD. MRU imaging offers superior resolution compared with ultrasound and provides all images of the kidneys and ureters from different panels at the same time, making it easier for clinical urologists to interpret the results. Therefore, in this study, we used the MRU-UUTD grading system instead of the Society for Fetal Urology (SFU) grading system, based on ultrasound for assessment and follow-up of NLUTD patients. Hydronephrosis and ureter dilation can be displayed in the same image of the three-dimensional reconstruction panel during a 360-degree rotation, and the UVJ stricture, ureter tortuosity, and stenosis can be displayed clearly. Typical imaging findings of oUUTD and roUUTD include external compression, stricture, and angulation of the ureter, with proximal ureter tortuosity and dilation above the obstruction. UUTO is confirmed by perfusion time-activity curves and the time from peak to 50% activity (T1/2) in isotope renography. Mechanical or adynamic obstruction can be further distinguished by diuresis experiment. Presurgical differential diagnosis of UUTD (UVR, UUTO, both, or neither) is essential, because the identification of mUUTO requires the procedures of urethroplasty. UVJ stenosis is an important factor in the etiology of mUUTO. Detrusor fibrosis, detrusor thickening, and poor bladder compliance secondary to progressive destruction of the bladder wall often result in ureteral stricture at the UVJ and proximal ureter dilation (4, 22). However, many patients exhibit a higher level of mUUTO, which is more frequent in this cohort (Figures 2, 3). It may be caused by retrograde flow of urine containing a large number of bacteria to the ureter and renal pelvis. Recurrent urinary tract infections can lead to inflammatory exudation, ureteritis, transmural scarring, and fibrous band formation in the retroperitoneal tissue, which contribute significantly to roUUTD's pathological process. High-level mUUTO may also occur without the presence of VUR, which may be explained by the vanishment of VUR with chronic disease progression.

Sometimes it is challenging to distinguish mUUTO from nonobstructive dilation. Isotope renography can assess the differential renal function and identify obstructions in an objective and reproducible way; however, the definition and parameters of mUUTO in NLUTD patients have not been well investigated (13). There are cases with typical imaging signs that failed to be diagnosed as obstruction on renography, and vice versa. mUUTO should be diagnosed according to comprehensive information, including clinical history, UUTD improvement after preoperative bladder drainage, MRU, and isotope renography.

Indeed, our research had limitations. First, we could not provide data to directly compare the efficacy of AUEC and traditional AC in patients with mUUTO owing to the clinical situation and medical ethics. However, the failure of sufficient preoperative bladder drainage may indicate that oUUTD or roUUTD cannot be relieved without ureteroplasty. Second, the isotope renography data for evaluating postsurgical differential renal function and obstruction was not acquired. Nevertheless, the UUTD was relieved and the typical obstruction on imaging resolved, indicating mobilization of the ureter. Third, the long-term follow-up data was limited. Due to the long follow-up period and the retrospective nature of this study, the results may have loss bias and information bias. Well-designed prospective studies with long follow-up time is needed to confirm the findings.

In this study, we analyzed a series of patients with intractable NLUTD characterized as oUUTD or roUUTD. All patients had a diagnosis of mUUTO according to preoperative bladder drainage, MRU, and isotope renography. It is not sufficient to perform AC and/or UARI. Therefore, we introduced a modified surgical procedure, AUEC, which concerns more the mobilization of the ureter and the necessity of ureteroplasty. Our results suggest the protective role on UUTD and renal function. A long-term follow-up study is necessary to confirm the findings.

## Data Availability

The raw data supporting the conclusions of this article will be made available by the authors, without undue reservation.
